# An Aggregation-Free Local Volume Fraction Formulation for Topological Design of Porous Structure

**DOI:** 10.3390/ma14195726

**Published:** 2021-09-30

**Authors:** Kai Long, Zhuo Chen, Chengwan Zhang, Xiaoyu Yang, Nouman Saeed

**Affiliations:** State Key Laboratory for Alternate Electrical Power System with Renewable Energy Sources, North China Electric Power University, Beijing 102206, China; michaelchak@126.com (Z.C.); m13956027314@163.com (C.Z.); yxyylc@foxmail.com (X.Y.); noumansaeed7846@gmail.com (N.S.)

**Keywords:** topology optimization, aggregation-free approach, augmented lagrangian method, porous structure design, infill structure

## Abstract

Cellular structure can possess superior mechanical properties and low density simultaneously. Additive manufacturing has experienced substantial progress in the past decades, which promotes the popularity of such bone-like structure. This paper proposes a methodology on the topological design of porous structure. For the typical technologies such as the p-norm aggregation and implicit porosity control, the violation of the maximum local volume constraint is inevitable. To this end, the primary optimization problem with bounds of local volume constraints is transformed into unconstrained programming by setting up a sequence of minimization sub-problems in terms of the augmented Lagrangian method. The approximation and algorithm using the concept of moving asymptotes is employed as the optimizer. Several numerical tests are provided to illustrate the effectiveness of the proposed approach in comparison with existing approaches. The effects of the global and local volume percentage, influence radius and mesh discretization on the final designs are investigated. In comparison to existing methods, the proposed method is capable of accurately limiting the upper bound of global and local volume fractions, which opens up new possibilities for additive manufacturing.

## 1. Introduction

Porous structures such as bones, coral and sponges have been widely discovered in nature. In recent years, the additive manufacturing (AM) technique provides tremendous opportunities to fabricate cellular structure with complicated shapes. As a powerful tool for innovative design, topology optimization brings the possibility of realizing novel properties beyond traditional materials with porous structure [1], e.g., architected material with programmable Poisson’s ratio [2,3], chiral metamaterial [4], bi-mode artificial meta-material [5] and lattice sandwich composite [6].

Along with the development of AM, much attention has been paid to the optimal design of multi-scale structures in the past few years [7]. In such a multi-scale design, also named concurrent design and hierarchical design, the macrostructure and material constructed by porous structures evolve simultaneously. Since a hierarchical optimization formulation was proposed by Rodrigues et al. [8], there is considerable literature in this aspect [9,10,11,12]. To facilitate the manufacturability, Liu et al. presented a mathematical model for concurrent design on the assumption that the identical microstructure is periodically distributed over the macrostructure [13]. Two separated subsystems, i.e., macrostructure and microstructure, are integrated into one unity by means of numerical homogenization. To realize the resultant design, the connectivity of microstructure needs to be taken into account. Li et al. suggested that the immediate density within a certain range can be classified into the same group [14]. Wang et al. proposed a shape metamorphosis concept with the purpose of generating a sequence of graded microstructures to ensure the connectability [15]. To address the connectivity issues, Liu et al. predefined connective areas in the periodic micro-scale units [16]. Li et al. developed a topology optimization approach for quasi-periodic microstructures by erode-dilate operations [17]. In the framework of the level set method, the variable cutting technique was introduced to obtain the cellular structures [18,19,20], which guarantees connections between the adjacent elements without extra constraints. The lattice structures can be also achieved by the moving morphable components approach [21,22]. Besides the homogenization theory, the substructure and a promising post-process named as de-homogenization can also be the candidate to bridge the macrostructure and porous material [23,24,25,26,27]. For the extensive topic, readers can be referenced to the insightful review [28].

Another way to generate the bone-like structures can be resorting to the porosity control, which may stem from the maximum size constraint in the topology optimization community. Guest and Prévost initially proposed a maximum length dimension restriction by requiring a minimum void volume in a neighborhood surrounding each voxel, in which a penalty term for the objective function and barrier function was introduced [29,30,31]. The maximum size was also integrated into the bi-directional evolutionary structural optimization method [32,33]. Wu et al. proposed infill formulation by imposing the local volume fraction (LVC) on the given region [34]. To handle with vast constraints, the p-norm aggregation function is employed to condense local constraints. Furthermore, this efficient scheme is subsequently extended to the structure containing interior infill wrapped with coated surface, self-supporting structure [35,36], heat transfer structure [37] and multiphase material structure [38]. Liang and Cheng proposed the sequential integer programming to directly solve bounds of linear infill constraints [39]. In the near term, Chen et al. presented a novel parameterized level set method for designing functionally graded cellular structure [40]. Lazarov and Wang presented a band-pass filter, which can result in a maximum size feature [41]. Dou provided an implicit control for local volume percentage by projection manipulation, including two filters followed by two projections and one multiplication [42]. In the case of the explicit and implicit control methods, an apparent defect is that the predefined local constraint cannot be exactly satisfied. Fernández et al. investigated the influence of the *p*-norm and *p*-mean function on the distribution and amount of data in the maximum size control [43]. Clausen et al. experimentally proved that the resistance to buckling for the infill structure can be substantially enhanced compared to structures from the traditional minimum compliance formulation [44].

Despite the typical aggregation formulation for porous structure being successfully performed in various engineering applications, the violation of the maximum LVC in existing literature is still a practical problem. In addition, the appropriate value for the aggregation parameter is also a challenge. More recently, the augmented Lagrangian (AL) method was successfully applied to handle millions of stress constraints [45,46,47,48]. Inspired by these facts, we reformulated the LVC constraint by virtue of the AL approach. In comparison with the existing method, the infill structures can be efficiently achieved with the accurate satisfaction of both global and local volume constraints.

The remainder of this paper is indicated as below. Section 2 describes topology optimization formulation for porous structure and the augmented Lagrangian method. Section 3 presents several numerical examples and discussions. Section 4 summarizes the conclusion.

## 2. Optimization Problem for Infill Structure

### 2.1. Formulation for Porous Structure Design

It is assumed that the structural design domain is partitioned into total *Ne* finite elements. The SIMP (Simplified Isotropic Material with Penalization) method is used in this study, with three fields [49]: the design variable field *ρ*, the filtered field $\tilde{\rho }$ and the projected field $\bar{\rho }$ The existence and null of the element are denoted by the projected field $\bar{\rho }$, which is linked to the design variable field *ρ* by

(1) $${\bar{\rho }}_{e}=\frac{\tanh\left(\varpi \theta \right)+\tanh\left(\varpi \left({\tilde{\rho }}_{e}-\theta \right)\right)}{\tanh\left(\varpi \theta \right)+\tanh\left(\varpi \left(1-\theta \right)\right)}$$

where *ϖ* and *θ* control the steepness and the threshold of the projection, respectively. Here ${\tilde{\rho }}_{e}$ is obtained through the density filtering process [50,51].

(2) $${\tilde{\rho }}_{e}=\frac{1}{\sum_{i\in N_{e,i}}\omega_{ei}}\sum_{i\in N_{e,i}}\omega_{ei}\rho_{i}$$

where *N_e_*_,*i*_ denotes the neighborhood set of elements that fall inside the *i*th element’s filter radius *r*_min_. The weighting factor *ω_ei_* is given as

(3) $$\omega_{ei}=\max\left(0,r_{\min}-\Delta \left(e,i\right)\right)$$

here Δ(*e*, *i*) represents the distance between the center the *e*th element and *i*th element.

The Yong’s modulus *E_e_* is parameterized by ${\bar{\rho }}_{e}$ according to the SIMP interpolation [52]:

(4) $$E_{e}=E_{\min}+{\bar{\rho }}_{e}^{\eta }\left(E_{0}-E_{\min}\right)$$

where *E*_0_ is the elastic modulus of solid material and *E*_min_(=10^−6^*E*_0_) is a minor stiffness to prevent singularity in finite element analysis. *η* denotes the power exponent of the Young’s modulus interpolation law, which is typically set as 3.

The local volume fraction can be defined as following [35]

(5) $${\bar{\bar{\rho }}}_{e}=\frac{\sum_{i\in M_{e,i}}{\bar{\rho }}_{i}}{\sum_{i\in M_{e,i}}1}$$

where *M_e_*_,*i*_ is the set of adjacent elements that fall inside the *i*th element’s circle or sphere within the influence radius *r*_max_.

Aiming to generate the porous structure automatically, the optimization problem can be mathematically formulated as pursuing the minimum compliance subject to local or/and global volume fraction constraints, i.e.,

(6) $$\begin{array}{l} \mathrm{find}\;\;\rho \\ \min\;\;c=f^{T}u\\ s.t.\;\;\;Ku=f\\ \;\;\;\;\;\;\;\;g_{e}={\bar{\bar{\rho }}}_{e}/\varphi -1\leq 0,\left(e=1,2,\ldots ,N_{e}\right)\\ \;\;\;\;\;\;\;\;g_{0}=V_{f}/\psi -1=\sum_{e=1}^{N_{e}}{\bar{\rho }}_{e}V_{e}/\psi \sum_{e=1}^{N_{e}}{\bar{\rho }}_{e}-1\leq 0\\ \;\;\;\;\;\;\;0\leq \rho_{e}\leq 1,\;\;\;\;\;\;\;\;\;\;\;\;\left(e=1,2,\ldots ,N_{e}\right) \end{array}$$

where *K*, *u* and *f* are the global stiffness matrix, displacement vector and external force vector, respectively. The Objective function is the static compliance *c*, which is adopted as the performance of structural stiffness. *V_f_* represents the global volume faction while *ψ* is its corresponding threshold. *V_e_* is the eth elemental volume of the solid material. The parameter *φ* denotes the local volume fraction for all voxels. It should be highlighted that the restriction on overall material usage is unnecessary since the local volume fraction is already specified. Under normal circumstances, the value of *ψ* needs to be satisfied with the condition *ψ* ≤ *φ* when the total volume fraction constraint is active.

### 2.2. Augmented Lagrangian Method

Currently, a variety of optimizers are available in the topology optimization community. For instance, the compliance minimization problems involving sole volume constraint can be effectively resolved using heuristic optimality criteria and rigorous mathematical programming, e.g., the method of moving asymptotes (MMA) [53] and sequential quadratic programming (SQP) [54,55]. The introduction of the Lagrange multiplier to satisfy the constraint equation is nothing new in the level set method (LSM) [56] and the Bi-directional Evolutionary Structural Optimization (BESO) method [57,58]. LSM and BESO do not account for a quadratic term in the constraint function introduced by the AL method. Furthermore, the AL method introduces considerably more constraint functions than the LSM and BESO methods. However, the AL method becomes appealing when a large scale of local stress constraints can be addressed successfully, according to a recent report [46]. In this work, the Equation (6) can be solved as the solution of the unconstrained program, by establishing a sequence of AL functions in each *k*-th step [47]:

(7) $$\underset{\rho_{e}\in \left[0,1\right]}{\min}\;\;\;\;ℜ\left(\rho ,\lambda^{\left(k\right)},\mu^{\left(k\right)}\right)=c+\frac{1}{N_{\Phi }}\Phi^{\left(k\right)}\left(\rho \right)+\frac{1}{N_{\Psi }}\Psi^{\left(k\right)}\left(\rho \right)$$

where *N_Φ_* and *N_Ψ_* are the constants related to number of the local and global volume constraints. The penalization terms *Φ*^(*k*)^(*ρ*) and *Ψ*^(*k*)^(*ρ*) are defined as follows [47].

(8) $$\Phi^{\left(k\right)}\left(\rho \right)=\sum_{e=1}^{N_{e}}\left[\alpha_{e}^{\left(k\right)}\beta_{e}\left(\rho \right)+\frac{\gamma_{}^{\left(k\right)}}{2}\beta_{e}{\left(\rho \right)}^{2}\right]$$

(9) $$\Psi^{\left(k\right)}\left(\rho \right)=\alpha_{0}^{\left(k\right)}\beta_{0}\left(\rho \right)+\frac{\gamma_{}^{\left(k\right)}}{2}\beta_{0}{\left(\rho \right)}^{2}$$

where the vector $\beta^{\left(k\right)}={\left[\beta_{0}^{\left(k\right)},\beta_{1}^{\left(k\right)},...,\beta_{N_{e}}^{\left(k\right)}\right]}^{T}$ can be rewritten as

(10) $$\beta^{\left(k\right)}\left(\rho \right)=\max\left[g\left(\rho \right),-\frac{\alpha_{e}^{\left(k\right)}}{\gamma_{}^{\left(k\right)}}\right]$$

In this work, we define $g={\left[g_{0},g_{1},...,g_{N_{e}}\right]}^{T}$ as the constraint vector containing both global and local volume constraint. The Lagrange multiplier vector $\alpha_{e}^{\left(k\right)}={\left[\alpha_{0}^{\left(k\right)},\alpha_{1}^{\left(k\right)},...,\alpha_{N_{e}}^{\left(k\right)}\right]}^{T}$ and penalty factor *γ*^(*k*)^ can be updated as following:

(11) $$\alpha_{}^{\left(k+1\right)}=\alpha_{}^{\left(k\right)}+\mu^{\left(k\right)}\beta \left(\rho^{\left(k\right)}\right)$$

(12) $$\gamma_{}^{\left(k+1\right)}=\min\left[\kappa \gamma_{}^{\left(k\right)},\gamma_{\max}\right]$$

where *κ*(>1) and *γ*_max_ are the update factor and the upper bound for *γ* respectively, with the adoption to ensure numerical stability.

### 2.3. Sensitivity Analysis

We will solve the Equation (7) based on the gradient information, and the sensitivity expressions for each term will be derived in this section. The sensitivity of the compliance *c* with respect to physical density ${\bar{\rho }}_{i}$ can be given as [53]:

(13) $$\frac{\partial c}{\partial {\bar{\rho }}_{i}}=-\eta {\bar{\rho }}_{i}^{\eta -1}u_{i}^{T}k_{0}u_{i}^{}\;$$

where *u_i_* and *k*_0_ are the displacement vector for *i*th element and local stiffness matrix when physical density ${\bar{\rho }}_{i}=1$.

The sensitivity of the penalization terms *Φ*^(*k*)^(*ρ*) and *Ψ*^(*k*)^(*ρ*) with respect to physical density ${\bar{\rho }}_{i}$ can be computed as

(14) $$\frac{\partial \Phi^{\left(k\right)}}{\partial {\bar{\rho }}_{i}}=\left(\alpha_{i}^{\left(k\right)}+\gamma_{}^{\left(k\right)}\beta_{i}\left(\rho \right)\right)\;\frac{\partial \beta_{i}\left(\rho \right)}{\partial {\bar{\rho }}_{i}}$$

(15) $$\frac{\partial \Psi^{\left(k\right)}}{\partial {\bar{\rho }}_{i}}=\left(\alpha_{0}^{\left(k\right)}+\gamma_{}^{\left(k\right)}\beta_{0}\left(\rho \right)\right)\;\frac{\partial \beta_{0}\left(\rho \right)}{\partial {\bar{\rho }}_{i}}$$

where $\partial \beta_{i}\left(\rho \right)/\partial {\bar{\rho }}_{i}=0$ when $g_{i}\left(\rho \right)<-\alpha_{i}^{\left(k\right)}/\gamma_{}^{\left(k\right)}$. The rest of the cases satisfy the following equation:

(16) $$\frac{\partial \Phi^{\left(k\right)}}{\partial {\bar{\rho }}_{i}}=\frac{\alpha_{i}^{\left(k\right)}+\gamma_{}^{\left(k\right)}\beta_{i}\left(\rho \right)}{\varphi }\sum_{j\in M_{j,i}}\frac{\partial {\bar{\bar{\rho }}}_{j}}{\partial {\bar{\rho }}_{i}}=\frac{\alpha_{i}^{\left(k\right)}+\gamma_{}^{\left(k\right)}\beta_{i}\left(\rho \right)}{\varphi }\sum_{j\in M_{j,i}}\frac{1}{\sum_{j\in M_{j,i}}1}$$

(17) $$\frac{\partial \Psi^{\left(k\right)}}{\partial {\bar{\rho }}_{i}}=\left(\alpha_{0}^{\left(k\right)}+\gamma_{}^{\left(k\right)}\beta_{0}\left(\rho \right)\right)\frac{V_{i}}{\sum_{i=1}^{N_{e}}{\bar{\rho }}_{i}}$$

According to the chain rule, the sensitivity of the function *ρ_e_* to design variable *ρ*_e_ can be written as

(18) $$\frac{\partial \left(\cdot \right)}{\partial \rho_{e}}=\sum_{i\in N_{i,e}}\frac{\partial \left(\cdot \right)}{\partial {\bar{\rho }}_{i}}\frac{\partial {\bar{\rho }}_{i}}{\partial {\tilde{\rho }}_{i}}\frac{\partial {\tilde{\rho }}_{i}}{\partial \rho_{e}}$$

where (·) can represent the individual function *c*, *Φ* and *Ψ* in Equation (7).

### 2.4. MMA Algorithm

In each optimization, the MMA approximation of the Equation (7) at the design variable vector *ρ*^(*k*)^ reads [48]:

(19) $$\underset{0\leq \rho_{e}\leq 1}{\min}:{\tilde{ℜ}}^{\left(k\right)}\left(\rho ,\alpha^{\left(k\right)}\right)=\sum_{e=1}^{N_{e}}\left(\frac{p_{e}^{\left(k\right)}}{U_{e}^{\left(k\right)}-\rho_{e}}+\frac{q_{e}^{\left(k\right)}}{\rho_{e}-L_{e}^{\left(k\right)}}\right)+r^{\left(k\right)}$$

where each individual term is expressed as:

(20) $$p_{e}^{\left(k\right)}={\left(U_{e}^{\left(k\right)}-\rho_{e}^{\left(k\right)}\right)}^{2}\left[\max\left(\frac{\partial {ℜ}^{\left(k\right)}}{\partial \rho_{e}},0\right)+10^{-3}\left|\frac{\partial {ℜ}^{\left(k\right)}}{\partial \rho_{e}}\right|+\frac{10^{-6}}{U_{e}^{\left(k\right)}-L_{e}^{\left(k\right)}}\right]$$

(21) $$q_{e}^{\left(k\right)}={\left(\rho_{e}^{\left(k\right)}-U_{e}^{\left(k\right)}\right)}^{2}\left[-\min\left(\frac{\partial {ℜ}^{\left(k\right)}}{\partial \rho_{e}},0\right)+10^{-3}\left|\frac{\partial {ℜ}^{\left(k\right)}}{\partial \rho_{e}}\right|+\frac{10^{-6}}{U_{e}^{\left(k\right)}-L_{e}^{\left(k\right)}}\right]$$

(22) $$r^{\left(k\right)}={\tilde{ℜ}}^{\left(k\right)}-\sum_{e=1}^{N_{e}}\left(\frac{p_{e}^{\left(k\right)}}{U_{e}^{\left(k\right)}-\rho_{e}}+\frac{q_{e}^{\left(k\right)}}{\rho_{e}-L_{e}^{\left(k\right)}}\right)$$

during the optimization iterations, the lower asymptote and upper asymptote can be determined as

(23) $$L_{e}^{\left(k\right)}=\rho_{e}^{\left(k\right)}-s_{e}^{\left(k\right)}\left({\overset{⌢}{\rho }}_{e}-{\underset{⌣}{\rho }}_{e}\right)$$

(24) $$U_{e}^{\left(k\right)}=\rho_{e}^{\left(k\right)}+s_{e}^{\left(k\right)}\left({\overset{⌢}{\rho }}_{e}-{\underset{⌣}{\rho }}_{e}\right)$$

where the parameter $s_{e}^{\left(k\right)}$ will be updated dynamically during the optimization iteration; The symbols ${\underset{⌣}{\rho }}_{e}$ and ${\overset{⌢}{\rho }}_{e}$ are the maximum and minimum value of design variable *ρ_e_*. When a positive move-limit *m* is predefined, it yields:

(25) $${\underset{⌣}{\rho }}_{e}^{}=\max\left(0,\rho_{e}^{\left(k\right)}-m\right)$$

(26) $${\overset{⌢}{\rho }}_{e}^{}=\min\left(1,\rho_{e}^{\left(k\right)}+m\right)$$

in the *k*th iteration, the design variables are made to satisfy the box constraint [48]:

(27) $$\max\left({\underset{⌣}{\rho }}_{e}^{},L_{e}^{\left(k\right)}+0.1\left(\rho_{e}^{\left(k\right)}-L_{e}^{\left(k\right)}\right)\right)\leq \rho_{e}^{\left(k\right)}\leq \min\left({\overset{⌢}{\rho }}_{e}^{},U_{e}^{\left(k\right)}-0.1\left(\rho_{e}^{\left(k\right)}-U_{e}^{\left(k\right)}\right)\right)$$

explicitly solving Equation (17) can be accomplished as follows.

(28) $$\rho_{e}^{new}=\max\left(\begin{array}{l} \max\left({\underset{⌣}{\rho }}_{e}^{},L_{e}^{\left(k\right)}+0.1\left(\rho_{e}^{\left(k\right)}-L_{e}^{\left(k\right)}\right)\right),\\ \min\left(\min\left({\overset{⌢}{\rho }}_{e}^{},U_{e}^{\left(k\right)}-0.1\left(\rho_{e}^{\left(k\right)}-U_{e}^{\left(k\right)}\right)\right),{ℑ}_{e}\right) \end{array}\right)$$

here, the ${ℑ}_{e}$ is found as

(29) $${ℑ}_{e}=\frac{L_{e}^{\left(k\right)}\sqrt{p_{e}^{\left(k\right)}}+U_{e}^{\left(k\right)}\sqrt{q_{e}^{\left(k\right)}}}{\sqrt{p_{e}^{\left(k\right)}}+\sqrt{q_{e}^{\left(k\right)}}}$$

## 3. Numerical Examples

In this section, several two-dimensional numerical tests are presented to prove the effectiveness of the proposed method. The material of the structure has Young’s modulus and Poisson’s ratio of 1 and 0.3. In all examples, the design domain was composed of the bilinear four-node elements. The projection parameter *θ* was fixed as 0.5 while *ϖ* started at 1 and was doubled per 50 steps until it reached the maximum 256. The move limit *m* was set as 0.05 to stabilize iterations. The examples in this paper were built on Matlab, and the program structure was based on this literature [59]. All examples were run on a PC with an Inter i7-9700K processor and 32 GB RAM.

### 3.1. Example 1

**Figure 1:**
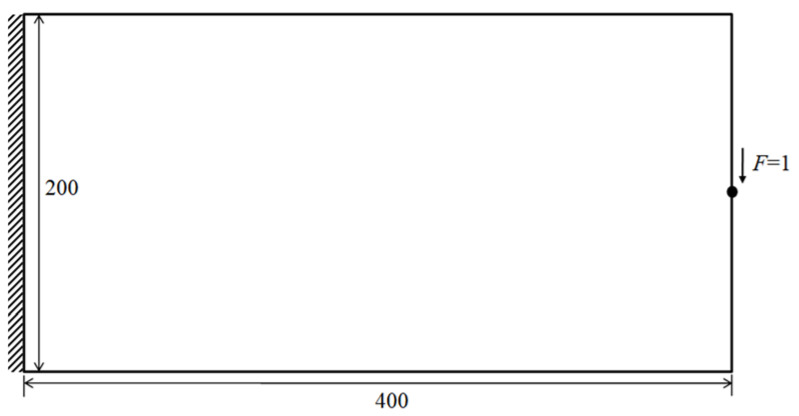
The design domain, boundary and loading condition for short cantilever structure.

**Figure 2:**
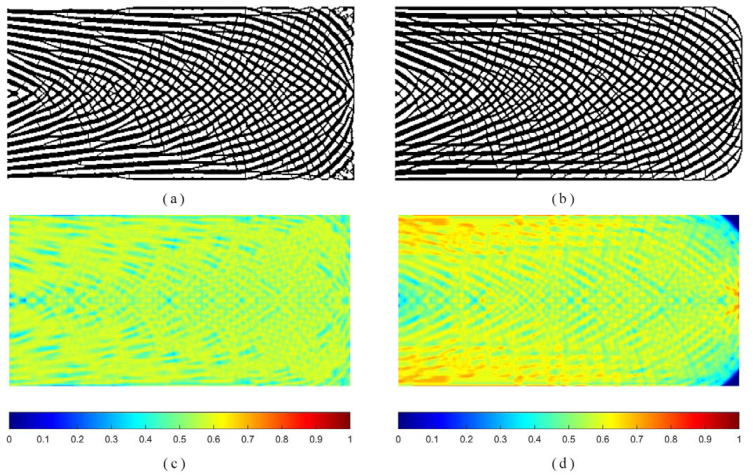
The optimized topologies obtained from: (**a**) the proposed method: *c* = 85.780, *V_f_* = 0.546; (**b**) *c* = 75.184, *V_f_* = 0.564 as well as the Local volume fraction distribution from: (**c**) the proposed method: $max\left({\bar{\bar{\rho }}}_{e}\right)=0.598$; (**d**) the *p*-norm method: $max\left({\bar{\bar{\rho }}}_{e}\right)=0.779$.

**Figure 3:**
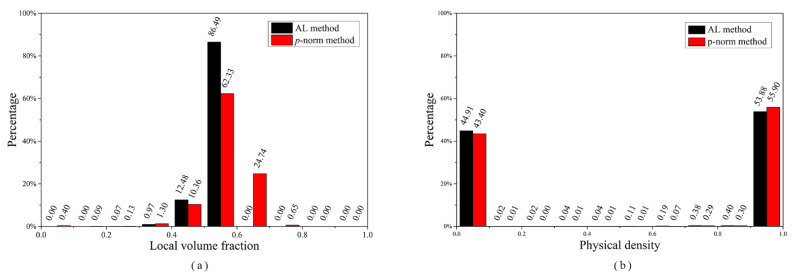
Histogram of (**a**) local volume fraction $\bar{\bar{\rho }}$; (**b**) physical density $\bar{\rho }$.

The first example optimizes a short cantilever as the benchmark in the existing literature [34]. Figure 1 shows a rectangular design domain, boundary and loading conditions. The overall dimensionless size of planar structure was: length *L* = 400 and height *H* = 200. The left side was fully supported, while a concentrated force was applied at the central point on the right edge. The design domain was subdivided into 400 × 200 elements. The local volume fraction and influence radius was set as *φ* = 0.6 and *r*_max_ = 6, respectively. For comparison purposes, the optimized topologies procured from the proposed method and the p-norm aggregation scheme and the distributions of the $\bar{\bar{\rho }}$ field are drawn in Figure 2, respectively. Additionally, the histograms of physical density $\bar{\rho }$ as well as local volume fraction $\bar{\bar{\rho }}$ are displayed in Figure 3.

From Figure 2, we can observe that the similar spongy-like structures can be obtained automatically via two approaches. The maximum of the local volume percentage 0.598 from the proposed method is slightly below the threshold value. Nevertheless, a serious violation of the local volume fraction occurs in the *p*-norm aggregation formulation. The visual differences in optimized topologies exist at two corners on the right side. Through the suggested method, the material is prone to be spread in the entire design area, which sounds reasonable in the absence of the total material usage. From the above phenomena, it is recommended that the global volume fraction is included in the proposed formulation.

From Figure 2, the local volume fraction distributions demonstrate that the proposed method is capable of achieving a uniform distribution of local volume fraction for each element, whereas the comparison method fails to control the local volume fraction in the structure’s critical-support region and load-bearing region. Furthermore, a more thorough analysis as shown in Figure 3a reveals that more than 80% of the elements in the proposed approach have a local volume fraction between 0.5 and 0.6, and none surpass the maximum limit of 0.6. Approximately 24% of elements have a local volume fraction that exceeds the upper limit as illustrated in Figure 3a. The primary reason for this discrepancy is that the suggested approach can precisely and directly restrict each constraint function, while the *p*-norm aggregation function roughly estimates the maximum value. Moreover, the physical density tends to a distinct 0–1 distribution as illustrated in Figure 3b.

### 3.2. Example 2

**Figure 4:**
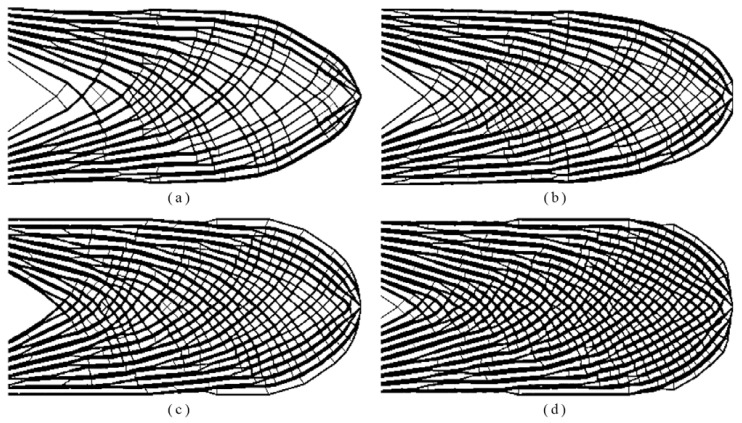
The optimized topologies by varying the global volume fraction from: (**a**) *ψ* = 0.35: *c* = 104.069, *V_f_* = 0.350, $max\left({\bar{\bar{\rho }}}_{e}\right)=0.595$; (**b**) *ψ* = 0.40: *c* = 94.551, *V_f_* = 0.401, $max\left({\bar{\bar{\rho }}}_{e}\right)=0.596$; (**c**) *ψ* = 0.45: *c* = 89.242, *V_f_* = 0.450, $max\left({\bar{\bar{\rho }}}_{e}\right)=0.595$; (**d**) *ψ* = 0.50: *c* = 87.399, *V_f_* = 0.502, $max\left({\bar{\bar{\rho }}}_{e}\right)=0.598$.

**Figure 5:**
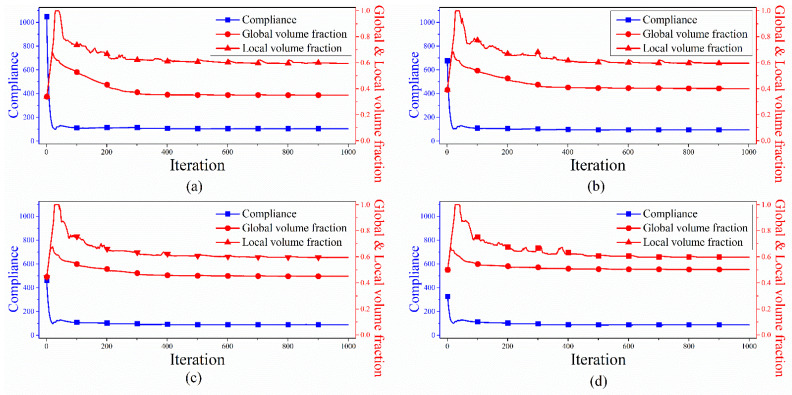
Iteration histories of compliance, global and local volume fraction from: (**a**) *ψ* = 0.35; (**b**) *ψ* = 0.40; (**c**) *ψ* = 0.45; (**d**) *ψ* = 0.50.

The influence of the global volume fraction on final design was investigated in this example. All parameters were identical with those in Example 1. Four upper bounds of the global volume fraction 0.35, 0.40, 0.45 and 0.50 were used, and the corresponding topologies were plotted in Figure 4. Figure 5 depicted the corresponding iterative curves of compliance, global volume fraction and maximum local volume fraction.

From Figure 4, we can see that a group of porous structures are generated by limiting the total material usages. Consistent with engineering intuition, the structure becomes much stiffer when more material is allowed in the design domain. Alongside this, we observed that the iteration curves exhibited a similar trend using various upper bounds of the global volume fraction as shown in Figure 5. The compliance decreased dramatically within the first 50 iterations while two curves of the global volume fraction and the maximum local volume fraction upsurged sharply, accompanied by the value of $\max\left({\bar{\bar{\rho }}}_{e}\right)$ reaching to 1. Then, all curves of the compliance underwent mild fluctuations and eventually reached the plateau. In summary, the material can be automatically and efficiently distributed to form a spongy structure by the proposed method meeting the requirement of the imposed constraints.

### 3.3. Example 3

**Figure 6:**
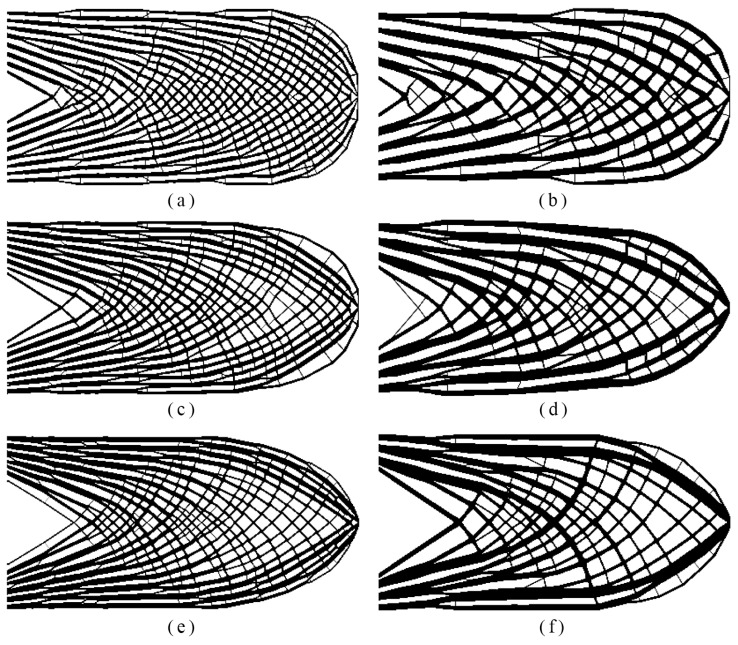
The optimized topologies obtained from (**a**) *φ* = 0.55, *r*_max_ =6: *c* = 99.577, *V_f_* = 0.449, $max\left({\bar{\bar{\rho }}}_{e}\right)=0.551$; (**b**) *φ* = 0.55, *r*_max_ = 12: *c* = 95.983, *V_f_* = 0.450, $max\left({\bar{\bar{\rho }}}_{e}\right)=0.546$; (**c**) *φ* = 0.65, *r*_max_ =6: *c* = 86.498, *V_f_* = 0.449, $max\left({\bar{\bar{\rho }}}_{e}\right)=0.645$; (**d**) *φ* = 0.65, *r*_max_ = 12: *c* = 82.466, *V_f_* = 450, $max\left({\bar{\bar{\rho }}}_{e}\right)=0.646$; (**e**) *φ* = 0.75, *r*_max_ =6: *c* = 78.921, *V_f_* = 0.451, $max\left({\bar{\bar{\rho }}}_{e}\right)=0.745$; (**f**) *φ* = 0.75, *r*_max_ = 12: *c* = 76.044, *V_f_* = 0.445, $max\left({\bar{\bar{\rho }}}_{e}\right)=0.746$.

**Figure 7:**
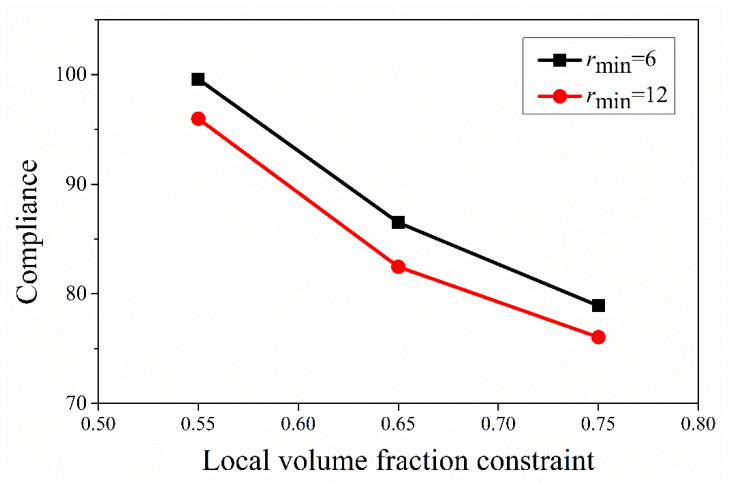
Relationship between *c*, *r*_max_ and *φ*.

In this numerical test, the effects of the local volume fraction constraint and influence radius *r*_max_ on optimized results are discussed. The global volume fraction was fixed as *ψ* = 0.45. The upper bound of the local volume fraction ranged from 0.55 to 0.70. Meanwhile two different influence radii *r*_max_ = 6 and 12 were adopted. The resulting topologies by various combinations of the parameter *φ* and *r*_max_ are listed in Figure 6. Figure 7 depicts the relationship between compliance, influence radius, and the local volume fraction constraint.

According to Figure 6, when the local volume fraction limit was relaxed, the number of pores in the porous structure decreased. Additionally, the larger influence radius *r*_max_ resulted in the following three effects: larger diameters for holes, fewer holes and stronger rods. As observed from Figure 7, the resultant structure became stiffer as the influence radius *r*_max_ and the upper bound of the local volume fraction *φ* grew. In conclusion, the local volume fraction constraint determined the local porosity, while the influence radius *r*_max_ played an important role on the void size of topology. These phenomena can be referenced as the design law for porous structures.

### 3.4. Example 4

**Figure 8:**
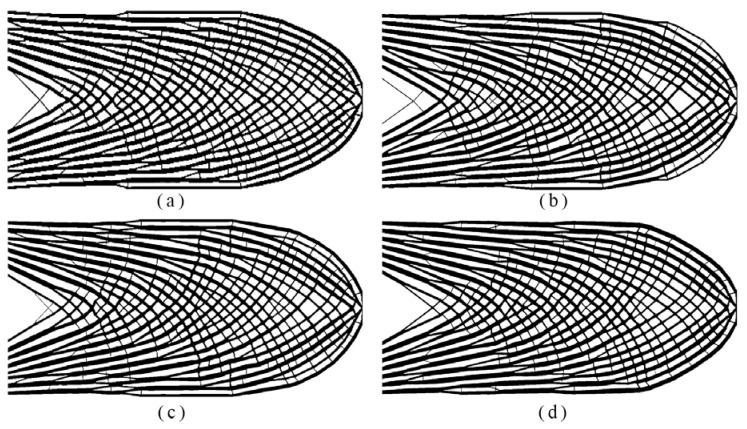
The optimized topologies from different meshes: (**a**) 400 × 200: *c* = 90.231, *V_f_* = 0.451, $max\left({\bar{\bar{\rho }}}_{e}\right)=0.597$; (**b**) 800 × 400: *c* = 88.148, *V_f_* = 0.449, $max\left({\bar{\bar{\rho }}}_{e}\right)=0.599$; (**c**) 1200 × 600: *c* = 86.557, *V_f_* = 0.446, $max\left({\bar{\bar{\rho }}}_{e}\right)=0.603$; (**d**) 1600 × 800: *c* = 86.208, *V_f_* = 0.448, $max\left({\bar{\bar{\rho }}}_{e}\right)=0.603$.

In addition to the work described in Example 3, which addressed the impact of the local volume fraction restriction and influence radius *r*_max_, a mesh-independence study was performed in Example 4. Four distinct meshes were utilized, including 400 × 200, 800 × 400, 1200 × 600 and 1600 × 800 elements. Two filtering radii and two volume fractions were fixed as *r*_min_ = 3, *r*_max_ = 6, *φ* = 0.6 and *ψ* = 0.45, respectively. The optimized topologies with the resulting compliance, volume fraction are displayed in Figure 8.

As seen from Figure 8, the analogous topologies were generated by different levels of discretization, albeit small discrepancies were noticed. For the densest elements case, more than one million local constraints were involved. Hence, it can be inferred that the proposed method can allow for efficient solution of the computationally demanding problem with mesh refinement.

### 3.5. Example 5

**Figure 9:**
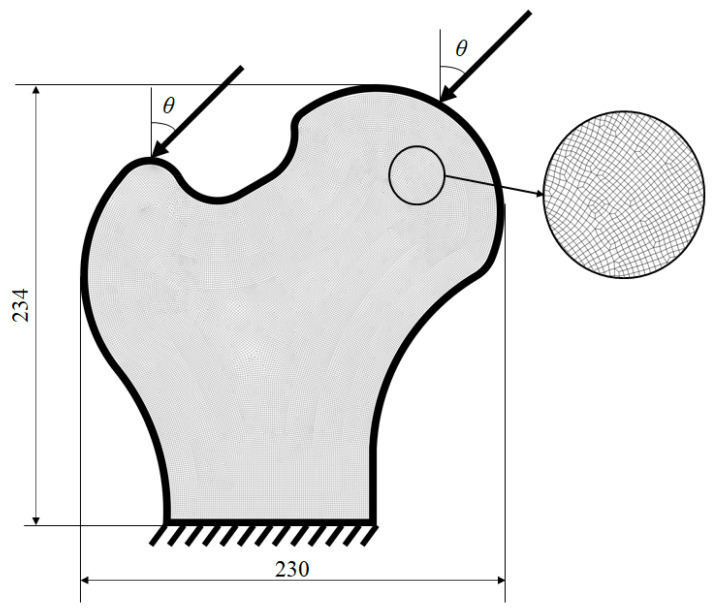
The design domain, boundary and loading condition for 2D bone-like structure.

**Figure 10:**
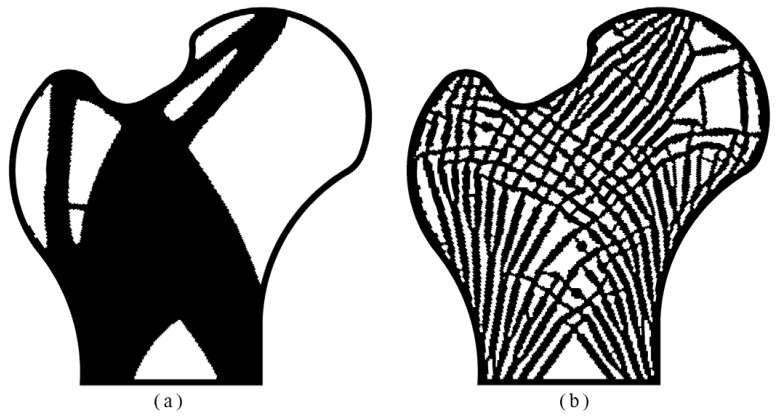
The optimized topologies (**a**) regardless of the local volume fraction: *c* = 2954.265, *V_f_* = 0.600, $max\left({\bar{\bar{\rho }}}_{e}\right)=1.000$; (**b**) by porosity control: *c* = 4658.439, *V_f_* = 0.595, $max\left({\bar{\bar{\rho }}}_{e}\right)=0.695$.

A 2D bone-like structure was optimized to exam the viability of the suggested method dealing with irregular shape and mesh. The finite element model consisted of 35,516 elements and 35,912 nodes, and four layers of elements were kept as the passive region. The bottom edge was entirely fixed while both the top left and top right corners were loaded diagonally downward at *θ* = 45° as illustrated in Figure 9. The magnitude of the left and right load was 2545 and 1697, respectively. The upper bounds for two volume fractions were set as *φ* = 0.7 and *ψ* = 0.6. For comparison, the topology optimization regardless of the local volume fraction was also performed. Two resultant topologies are displayed in Figure 10.

As shown in Figure 10, the optimized topologies reflect visual differences, i.e., the material accumulation regardless of the local volume proportion and real cross-section of the bone by porosity control.

**Figure 11:**
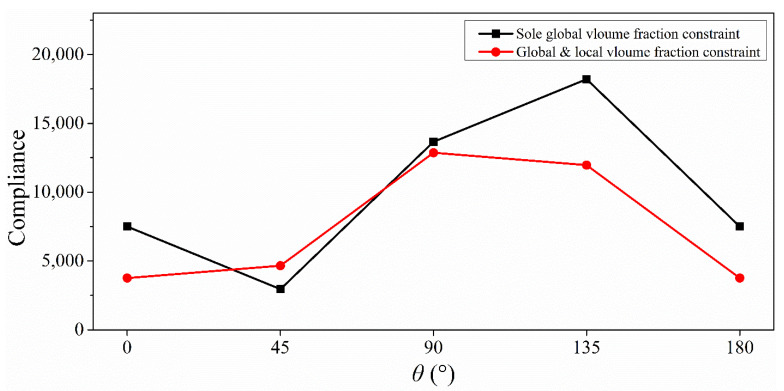
Compliance curves with respect to load direction angle *θ* of two resulting topologies.

The robustness of the final designs concerned to the force direction was verified. The resultant compliance of the bone-like structure sustaining loads in different directions were computed and are plotted in Figure 11. The compliance fluctuates substantially within the scope of the investigation by employing the traditional stiffness optimization. Comparatively, the curve from the suggested method underwent a relatively gradual shift. The optimized results clearly demonstrate that the bone-like structure is insensitive to the direction of external load.

## 4. Discussion and Conclusions

This paper proposed an alternative approach to generate the porous structure. Innovatively, masses of local volume fraction constraints were appended to the objective function. The optimum in the primary problem can be obtained by dividing into an array of sub-problems in the framework of the augmented Lagrangian function. The MMA approximation and solution at each iteration were conducted. The performance of the proposed method was numerically studied in detail, and its effectiveness to produce the infill structure was demonstrated by five 2D numerical examples.

It was found that the vast majority of the predefined LVCs can be strictly satisfied, which can be viewed as a remarkable advantage superior to previous methods that are unable to control the LVCs precisely. In addition, the upper bound of the local volume fraction and the influence radius synthetically determined the local porosity and void size. The proposed method can also provide solutions independent of discretization and insensitive to variation of the external force direction. In future work, the nonlinear or transient topology optimization problem will be furthered based on the current study.

## Data Availability

Data sharing not applicable.
